# Florigen Activation Complex Dynamics and SVP‐Mediated Repression Orchestrate Temperature‐Regulated Flowering in Saffron

**DOI:** 10.1111/pbi.70361

**Published:** 2025-10-13

**Authors:** Diksha Kalia, Joel Jose‐Santhi, Firdous Rasool Sheikh, Rajesh Kumar Singh

**Affiliations:** ^1^ Plant Adaptation and Developmental Biology Lab, Biotechnology Division CSIR‐Institute of Himalayan Bioresource Technology Palampur India; ^2^ Academy of Scientific and Innovative Research (AcSIR) Ghaziabad India

**Keywords:** bZIP transcription factor‐FD, floral induction, Florigen Activation Complex, flowering locus T, geophytes, phosphatidyl ethanolamine binding protein, *TFL1*, thermoperiodic, thermoresponsive, VIGS

## Abstract

Saffron, a high‐value spice cultivated worldwide for its therapeutic and culinary uses, is a sterile triploid species, rendering conventional breeding approaches ineffective. This limitation underscores the need for molecular and biotechnological strategies for its genetic improvement. Flowering, a key determinant of saffron yield, is strongly influenced by temperature; however, the genetic regulatory networks underlying this process remain poorly understood. Our study identifies key regulators of saffron's flowering, focusing on the Florigen Activation Complex (FAC) components: *FLOWERING LOCUS T* (FT), bZIP transcription factor‐FD and *TERMINAL FLOWER*‐1 (TFL‐1) and demonstrates their temperature‐dependent roles in floral regulation. Spatiotemporal expression analyses suggested that *CsatFT3* and *CsatFD2*, expressed in the floral meristem, promote floral induction, while *CsatTFL1*‐3 acts as a floral repressor. Protein interaction studies showed that CsatFT3 and CsatTFL1‐3 compete for binding to CsatFD2, and their balance modulates floral induction. Functional validation in Arabidopsis and Saffron confirmed these findings. Furthermore, we identified *CsatSVP2*, an ortholog of *SHORT VEGETATIVE PHASE* (*SVP*), as a low temperature‐responsive repressor that directly binds the CsatFT3 promoter to inhibit its expression. Together, these findings enhance our understanding of temperature‐mediated floral induction in saffron and provide insights and lay the groundwork for genetic interventions to enhance yield under variable temperature conditions.

## 1 Introduction

Saffron ( *Crocus sativus* L.) is a sterile, clonally propagated, autotriploid geophytic monocot cultivated for its highly valued dried stigmas, which constitute the world's most expensive spice. While the stigmas are the primary edible and culinary component, other parts of the saffron plant also have diverse applications, including uses in agriculture, traditional medicine and as a natural colouring agent, making the entire flower economically valuable (Ahrazem et al. 2015). Saffron has been predominantly cultivated for centuries in select regions such as Iran, India, Greece, Italy, Afghanistan, Morocco, Spain and various Mediterranean basins (Cardone et al. 2020). Flowering is the key determinant of saffron's crop productivity, and this critical phase is increasingly influenced by environmental changes. As a thermoperiodic plant, saffron's flowering is primarily regulated by temperature. During the warm summer months, the transition from vegetative to reproductive growth occurs underground, with floral bud emergence only triggered by exposure to cooler temperatures (Molina, Valero, Navarro, Guardiola, and Garcia‐Luis 2005). This biphasic thermal requirement consists of an induction phase at warm temperatures lasting 50–150 days, followed by an emergence phase under cooler conditions, a pattern that has been well documented (Jose‐Santhi et al. 2023; Molina, Valero, Navarro, Guardiola, and Garcia‐Luis 2005). The inappropriate temperature during the flowering transition leads to flower atrophy or no flower, causing yield loss (Wang et al. 2021). Saffron corms can sense the temperature change and modify the response accordingly (Molina, Valero, Navarro, Garcia‐Luis, and Guardiola 2005). Thus, there exists a thermoresponsive regulation of flowering in saffron, which is still not well studied at the molecular level.

Flowering is a key transition in the angiosperm life cycle, marking the shift from vegetative to reproductive growth and representing a tightly regulated process vital for survival. This process is orchestrated by a complex network of environmental cues, internal signalling pathways, hormonal regulation and genetic programmes that together ensure flowering occurs under optimal conditions (Zik and Irish 2003). Key environmental pathways influencing flowering include photoperiod, ambient temperature, light quality, vernalisation and gibberellin signalling. These pathways operate in coordination with endogenous factors along with plant age and nutritional status, allowing plants to synchronise reproduction with favourable environmental windows (Andrés and Coupland 2012; Freytes et al. 2021; Pyo et al. 2014; Srikanth and Schmid 2011). Extensive studies in both monocot and dicot organisms, such as *Arabidopsis thaliana* and rice, have uncovered key regulatory components of flowering (Kinoshita and Richter 2020). Central to this regulation are floral integrator genes, particularly *FLOWERING LOCUS T* (*FT*) in Arabidopsis and *Heading Date 3a* (*Hd3a*) in rice, which belong to the phosphatidylethanolamine‐binding protein (PEBP) family. FT/Hd3a promotes flowering by forming the Florigen Activation Complex (FAC), composed of FD‐like bZIP transcription factors and the florigen receptor 14–3‐3 protein (Abe et al. 2005; Taoka et al. 2013). This complex activates floral meristem identity genes such as *LEAFY* and *APETALA1*, initiating floral development (Putterill et al. 2004; Simon et al. 1996; Wigge et al. 2005). In contrast, *TERMINAL FLOWER 1* (TFL1), another PEBP family member, acts antagonistically to repress flowering (Wickland and Hanzawa 2015). In rice, Hd3a, RFT1 and OsFTL10 promote flowering by forming the Florigen Activation Complex (FAC) (Komiya et al. 2008; Tamaki et al. 2007). Conversely, OsFTL12, OsFTL4, RCN1 and RCN2 act as floral repressors by competing for FAC components to assemble a Florigen Repression Complex (FRC) (Nakagawa et al. 2002; Zheng et al. 2023). The dynamic balance between FAC and FRC finely regulates heading date and plant architecture in rice. Members of the FD family are key interaction partners of the PEBP family, playing roles in both promoting and repressing meristem differentiation, depending on their specific interacting partners (Zhu et al. 2020). Concurrently, the flowering program suppresses negative regulators, including *FLOWERING LOCUS C* (*FLC*) and *SHORT VEGETATIVE PHASE* (*SVP*), which are crucial for ensuring a proper transition from the vegetative to the reproductive phase mainly regulated by temperature (Bowman et al. 2012; Turck et al. 2008). Temperature‐dependent flowering has been widely studied, with FT‐like genes playing a central role (Capovilla et al. 2015). Upstream regulators include MADS‐domain transcription factors such as SVP and FLC, which modulate flowering by forming complexes and repressing *FT* expression via direct binding to its promoter (Balasubramanian et al. 2006; Gu et al. 2013; Lee et al. 2007). However, the thermoresponsive pathway governing floral induction in saffron remains largely unexplored.

In contrast to annuals, the regulation of flowering in perennial geophytes involves modified or additional mechanisms to accommodate their extended life cycles, periods of dormancy, and dual reproduction (Khosa et al. 2021). This has been observed in species like *Narcissus tazetta* (Noy‐Porat et al. 2013), tulip (Tulipa spp.) (Leeggangers et al. 2017) and saffron (Molina, Valero, Navarro, Guardiola, and Garcia‐Luis 2005), where floral induction occurs even in the absence of leaves. In these species, flowering is thought to be mediated directly at the meristem level, independent of traditional photoperiod or vernalisation cues. Instead, ambient temperature plays a dominant role in determining flowering time. These observations highlight the need to identify the specific genes and mechanisms that govern flowering in geophytic perennials. Over evolutionary time, the molecular mechanisms controlling flowering have grown increasingly complex, involving intricate interactions between floral regulator genes and their transcription factor partners, which form regulatory complexes (Wickland and Hanzawa 2015). Gene duplication and neofunctionalisation have contributed to the diversification of these regulatory genes. Both *FT* and *TFL1*, the central regulators of flowering, have undergone gene duplication events in various lineages, leading to paralogs with distinct functions that promote or inhibit flowering (Jin et al. 2021). This evolutionary diversification is evident across many species. In geophytes, for instance, members of the PEBP gene family have extended their functions beyond flowering to include the formation of underground storage organs, contributing to both sexual and asexual reproduction (Khosa et al. 2021). In species like potato, tulip and others, gene duplication has led to distinct roles in flowering, vegetative growth and tuber or bulb formation (Bellinazzo et al. 2025; Jing et al. 2023; Navarro et al. 2011). Similar divergence has been observed in onion ( *Allium cepa* ), sugar beet ( *Beta vulgaris* ) and hybrid aspen, demonstrating the complexity of flowering regulation in perennials (Hsu et al. 2011; Lee, Baldwin, et al. 2013; Pin et al. 2010). Beyond the PEBP gene family, functional diversification is seen in related pathways. In hybrid aspen, two FD homologues, *FDL1* and *FDL2*, regulate seasonal growth differently (Tylewicz et al. 2015). In rice, *OsFD2* contributes to leaf development, while *OsFD1* regulates flowering (Tsuji et al. 2013). FD‐like transcription factors can have dual roles, promoting or repressing flowering depending on their partners. For instance, *CmFDa* in *Chrysanthemum morifolium* represses flowering through epigenetic regulation (Xue et al. 2025). These examples highlight the evolutionary plasticity of flowering networks and the importance of species‐specific regulatory mechanisms.

Saffron, due to its sterile nature and extremely limited genetic diversity, is not amenable to conventional breeding approaches, which hinders efforts to develop climate‐resilient, high‐yielding and stress‐tolerant varieties. As a result, there have been no significant advancements in improving saffron's adaptability to climate change, making its cultivation increasingly vulnerable to environmental fluctuations. Consequently, only biotechnological interventions can be employed to introduce desirable traits. Despite its economic and agricultural importance, research on the molecular regulation of flowering in saffron remains limited. Most existing studies have focused on temperature effects on flowering physiology (Molina, Valero, Navarro, Guardiola, and Garcia‐Luis 2005; Wang et al. 2021), and while several flowering‐related genes have been identified through transcriptomic and genomic analyses (Hu et al. 2020; Kalia et al. 2022; Renau‐Morata et al. 2021; Singh et al. 2023; Tsaftaris et al. 2013), these remain largely descriptive and lack functional validation. Expression profiling has indicated the involvement of multiple *FT* and *TFL1* homologues in saffron flowering (Kalia et al. 2022; Renau‐Morata et al. 2021; Tsaftaris et al. 2013), yet no functional characterisation of these genes has been reported. To fill this gap, we present a comprehensive molecular framework for temperature‐dependent floral induction in saffron, a geophytic monocot with a unique flowering phenotype. By integrating gene expression profiling, heterologous functional assays, protein–protein interaction studies, promoter binding analyses and virus‐induced gene silencing, we identify key regulatory components that mediate the floral transition in response to ambient temperature. Overall, these findings advance our understanding of temperature‐mediated flowering in saffron and provide a foundation for genetic interventions aimed at improving yield under fluctuating environmental conditions.

## 2 Methods

### 2.1 Plant Materials and Growth Conditions

Saffron corms ( *C. sativus* L.) were grown under controlled conditions at CSIR‐IHBT, and samples were collected monthly from January to September, covering both the vegetative growth and flowering phases (Jose‐Santhi et al. 2023; Kalia et al. 2022). To analyse gene expression patterns, apical buds, axillary buds and corm tissues were harvested across this period, with three biological replicates per time point, each comprising pooled tissue from ten individual corms. For RT‐qPCR analysis, apical buds, axillary buds, and corm tissue collected during the vegetative and flowering stages were used, with detailed methods and results provided in the Supporting Information.

ViGS experiments were conducted using corms grown under the same controlled conditions as described earlier. Corms with high flowering competency (weighing > 10 g) were selected for the silencing of *CsatFT3* and *CsatFD2*. To investigate the role of *CsatSVP2*, two experimental conditions were used: (i) standard growth conditions and (ii) a temperature‐sensitive treatment in which corms were incubated at 8 C during the flowering phase to assess temperature‐dependent gene regulation. For silencing of *CsatTFL1‐3/CEN1*, corms with lower flowering competency (weighing 7–9 g) were utilised.

*A. thaliana* (Col‐0) was used as the wild‐type background for transformation experiments, and the ft1 mutant in the Ler background was used for functional complementation of CsatFT3. *Nicotiana benthamiana* was used for transient expression assays. Arabidopsis seeds were surface‐sterilised with 4% sodium hypochlorite and germinated on half‐strength Murashige and Skoog (MS) medium. To synchronise germination, seeds were stratified at 4°C for 2 days before being transferred to a soil mixture of peat, vermiculite and perlite (2:1:1). All plants were cultivated in controlled growth chambers under long‐day conditions (16 h light/8 h dark) at 22°C for Arabidopsis and 25°C for *N. benthamiana*.

### 2.2 RNA Isolation and Expression Analysis by RT‐qPCR

Total RNA was isolated from saffron apical buds, axillary buds, corm tissues and Arabidopsis using the Plant Total RNA Isolation Kit (Sigma‐Aldrich), according to the manufacturer's protocol. For each sample, 1 μg of total RNA was used to synthesise cDNA. Reverse transcription was performed using the RevertAid cDNA Synthesis Kit. The transcript level of each gene was quantified by quantitative real‐time PCR (qPCR) using gene‐specific primers (Table S1), generated by SnapGene Viewer 5.3.1 software (version 0.4.0) using the system (Bio‐Rad CFX Opus Real‐time PCR). Each expression analysis included three biological replicates, with each replicate derived from pooled tissue of ten individual corms. RT‐qPCR results are presented as the mean ± standard error (SEM) of three technical replicates per biological sample.

### 2.3 Sequence Alignments and Phylogenetic Analysis

Protein sequences were aligned using the ClustalW algorithm with default parameters to generate high‐quality multiple sequence alignments. Visualisation of the alignments was performed using ESPript 3.x (Robert and Gouet 2014) to highlight conserved regions and structural features. The aligned sequences were exported in Newick (.nwk) format for phylogenetic analysis. Phylogenetic tree construction was carried out using the “build” function of ETE3 v3.1.3 (Huerta‐Cepas et al. 2016), implemented via the GenomeNet platform (https://www.genome.jp/tools/ete/). FastTree v2.1.8 (Price et al. 2010) was used with default settings to infer approximate maximum‐likelihood phylogenetic trees.

### 2.4 Plant Transformation

To generate Arabidopsis transgenic lines, full‐length coding sequences (CDS) of the target genes were amplified and first cloned into the pENTR entry vector using Gateway cloning technology. Subsequently, LR recombination was performed to transfer the CDS into the destination vector pK2GW7 (for overexpression analysis), under the control of the Cauliflower Mosaic Virus (CaMV) 35S promoter. The resulting constructs included 35S::*CsatFD1*, 35S::*CsatFD2*, 35S::*CsatFD3*, 35S::*CsatFT3*, 35S::*CsatTFL1‐1*, 35S::*CsatTFL1‐2*, 35S::*CsatTFL1‐3*, 35S::*CsatSVP1*, and 35S::*CsatSVP2*. Primer sequences used for vector construction are provided in the Table S1 file. The final recombinant plasmids were introduced into *A. thaliana* (Col‐0) via *Agrobacterium tumefaciens* strain GV3101 using the floral dip transformation method. Transgenic seeds were harvested individually and screened based on the appropriate selection marker. Homozygous T3 lines were identified and used for further phenotypic characterisation. Flowering time was recorded as the number of days from germination to the emergence of the first floral bud.

### 2.5 Yeast One Hybrid (Y1H) Assay

To examine the binding of *CsatSVP2* to the *CsatFT3* promoter, yeast one‐hybrid (Y1H) assays were conducted using the Matchmaker Gold Yeast One‐Hybrid System Kit (Clontech). Promoter fragments of *CsatFT3* containing the core CArG‐box motif were cloned into the pAbAi vector. The resulting recombinant pAbAi‐promoter plasmids were linearised with BstBI (NEB) and integrated into the genome of the Y1HGold yeast strain. Transformants were selected on synthetic dextrose (SD) medium lacking uracil (SD/‐Ura). The coding sequence of *CsatSVP2* was amplified and inserted into the pGADT7‐AD vector. The recombinant pGADT7‐AD constructs were then introduced into the Y1HGold strains containing the integrated *CsatFT3* promoter. Protein–DNA interaction was assessed by culturing the transformed yeast on SD medium lacking leucine (SD/−Leu) and supplemented with Aureobasidin A (AbA) at a minimal inhibitory concentration of 150 ng/mL for four days. Primer sequences used for cloning are provided in Table S1.

### 2.6 Histochemical Localisation of GUS Activity

GUS activity was performed using the histochemical staining protocol described by Jefferson (Jefferson et al. 1987). Transgenic *A. thaliana* lines with the *ProCsatFT3::GUS* construct were incubated in a staining solution composed of 50 mM sodium phosphate buffer (pH 7.2), 2.0 mM potassium ferricyanide [K_3_Fe(CN)_6_], 2.0 mM potassium ferrocyanide [K_4_Fe(CN)_6_], 0.1% (v/v) and 1.0 mg/mL X‐Gluc (5‐bromo‐4‐chloro‐3‐indolyl‐β‐D‐glucuronide). Samples were incubated at 37°C for 5 h to allow GUS expression to develop. Following staining, tissues were cleared with 70% (v/v) ethanol to remove chlorophyll and enhance contrast, then examined under a stereo microscope (ZEISS Stemi 508). To validate GUS expression patterns, at least 10 independent transgenic lines were screened for the construct. Plants from three confirmed GUS‐positive lines were selected for detailed expression analysis at various developmental stages, including 7, 14, and 21 days after germination (DAG), inflorescence emergence, flowering and silique formation. For the flowering and silique stages, staining was performed on aerial tissues from soil‐grown plants.

### 2.7 Yeast Two‐Hybrid (Y2H) Assays

Yeast two‐hybrid assays were conducted following the manufacturer's protocol using the Matchmaker Two‐Hybrid System (Clontech). The CDS of *CsatFD1*, *CsatFD2* and *CsatFD3* were cloned into the bait vector pGBKT7, whereas those of *CsatFT3*, *CsatTFL1‐1*, *CsatTFL1‐2* and *CsatTFL1‐3* were inserted into the prey vector pGADT7. The primers used for amplification are provided below. Bait and prey plasmids were co‐transformed into the Y2HGold yeast strain and cultured on synthetic dextrose (SD) medium lacking tryptophan (SD/−Trp) and leucine (SD/−Leu) for 3–5 days at 30°C. Protein–protein interactions were assessed by selecting colonies on SD medium lacking Trp, Leu and histidine (SD/−Trp/−Leu/‐His).

### 2.8 Bimolecular Fluorescence Complementation (BiFC)

The CDS of *CsatFD1*, *CsatFD2* and *CsatFD3* were cloned into YFP C‐terminal (YFPC), and *CsatFT3*, *CsatTFL1‐1*, *CsatTFL1‐2* and *CsatTFL1‐3* were cloned into the YFP N‐terminal (YFPN) vectors. Primer sequences used for vector construction are provided in the Table S1. These constructs were then introduced into *A. tumefaciens* strain GV3101 for transient expression in 5‐week‐old *N. benthamiana* leaves. After co‐infiltration for 48 h, fluorescence was imaged using a confocal laser scanning microscope Leica STELLARIS 5 (Leica Microsystems). YFP fluorescence was excited at 514 nm, with emission detected between 522 and 560 nm.

### 2.9 Firefly Complementation Assay

The full‐length CDSs of *CsatFT3*, *CsatTFL1‐3* and *CsatFD2* were cloned into the pCAMBIA1300‐nLUC and pCAMBIA1300‐cLUC vectors, respectively, and introduced into *A. tumefaciens* strain GV3101. Various combinations were co‐infiltrated into *N. benthamiana* leaves. The infiltrated plants were incubated at 22 C for 2 days. Prior to LUC activity detection, the leaves were sprayed with 1 mM D‐luciferin (potassium salt, BioVision) and incubated in the dark for 8 min to eliminate background fluorescence. Fluorescence was detected using a cooled CCD camera system (Bio‐Rad Chemi Doc). Relative LUC intensity was quantified using ImageJ software. Statistical analysis was performed using one‐way ANOVA, and significance was assessed using the Compact Letter Display (CLD) method to indicate differences at a significant *p*‐value level. Primer sequences used in these assays are provided in Table S1.

### 2.10 Genome Walker Library for Promoter Amplification

The 5′ upstream region of the *CsatFT3* gene from *C. sativus* was obtained using the Genome Walker kit (Clontech, USA). Genomic DNA was extracted from leaf tissue following the protocol provided with the QIAGEN DNA isolation kit, then digested with two blunt‐end restriction enzymes, DraI and EcoRV, and further ligated with adapters to generate Genome Walker libraries. Gene‐specific primers (GSPs), designed based on the *CsatFT3* cDNA sequence, were used alongside adaptor primers from the kit to carry out primary and nested PCR amplifications as listed in Table S1. The amplified PCR products were resolved on a 1% (w/v) agarose gel, purified and cloned into the pGEM‐T Easy vector (Promega, USA) for sequencing. The resulting *CsatFT3* promoter sequence was analysed using the PlantCARE database (Lescot et al. 2002) to identify potential cis‐regulatory elements involved in transcriptional regulation.

### 2.11 Virus‐Induced Gene Silencing (VIGS)

Virus‐induced gene silencing (VIGS) was carried out following the protocol established in the lab (Kalia et al. 2024). Gene‐specific fragments were amplified for *CsatFT3* (300 bp, flanked by EcoRI sites), *CsatFD2* (292 bp, flanked by BamHI sites), *CsatTFL1‐3* (302 bp, containing both EcoRI and BamHI sites) and *CsatSVP2* (318 bp, with EcoRI and BamHI sites) restriction sites at both termini. These fragments were cloned into the pTRV2 vector to generate the constructs. Agrobacterium‐mediated infiltration was performed as previously described (Kalia et al. 2024). To confirm gene silencing, transcript levels of *CsatFT3*, *CsatFD2*, *CsatTFL1‐3* and *CsatSVP2* were quantified by RT‐qPCR in apical buds collected at the floral induction stage. Phenotypic analysis of flower development was conducted when floral buds reached the floral emergence stage. Primer sequences used for the VIGS assay are listed in Table S1.

### 2.12 Luciferase Assay

To investigate the regulatory effect of *CsatSVP2* on the *CsatFT3* promoter, a luciferase reporter assay was conducted in *N. benthamiana* leaves. Promoter sequences of *CsatFT3* were cloned into the Gateway‐compatible vector pGWB435 (Invitrogen) upstream of the luciferase (Luc) reporter gene. Three promoter constructs were generated: (i) the full‐length promoter (FL), (ii) a truncated fragment containing the CArG motif(T1) and (iii) a truncated fragment lacking the CArG motif (T2). The *CsatSVP2* coding sequence was separately cloned into pGWB402 under the control of the CaMV35S promoter to serve as an effector. Control treatments included empty vectors (pGWB435 and pGWB402) as a negative control and *ProCsatFT3*::Luc alone as a positive control. Constructs were introduced into the *A. tumefaciens* strain GV3101 and co‐infiltrated into *N. benthamiana* leaves. Each leaf was divided into four quadrants to enable side‐by‐side comparisons of different treatments within the same biological context. Luciferase activity was visualised and quantified using ImageJ software. The experiment was repeated independently at least three times in separate leaves to ensure reproducibility. One‐way ANOVA was used for statistical analysis, and differences among treatments were determined using CLD to indicate statistically significant differences (*p* < 0.05).

### 2.13 Statistical Analyses

All statistical analyses were conducted using GraphPad Prism version 10 for Windows (GraphPad Software, Boston, MA, USA; www.graphpad.com). One‐way ANOVA followed by Tukey's Honest Significant Difference (HSD) test was applied for multiple comparisons. Statistically significant differences between groups at *p* < 0.05 are indicated by different letters, as determined by CLD. Two‐way ANOVA to assess the effects of temperature, month and their interaction, followed by Sidak's multiple comparisons test and Student's *t*‐test for one‐to‐one comparison. (*p < 0.05; **p < 0.01; ***p < 0.001; ****p < 0.0001).

## 3 Results

### 3.1 *CsatFT3* Functions as a Temperature‐Sensitive Regulator of Floral Induction in Saffron

**FIGURE 1:**
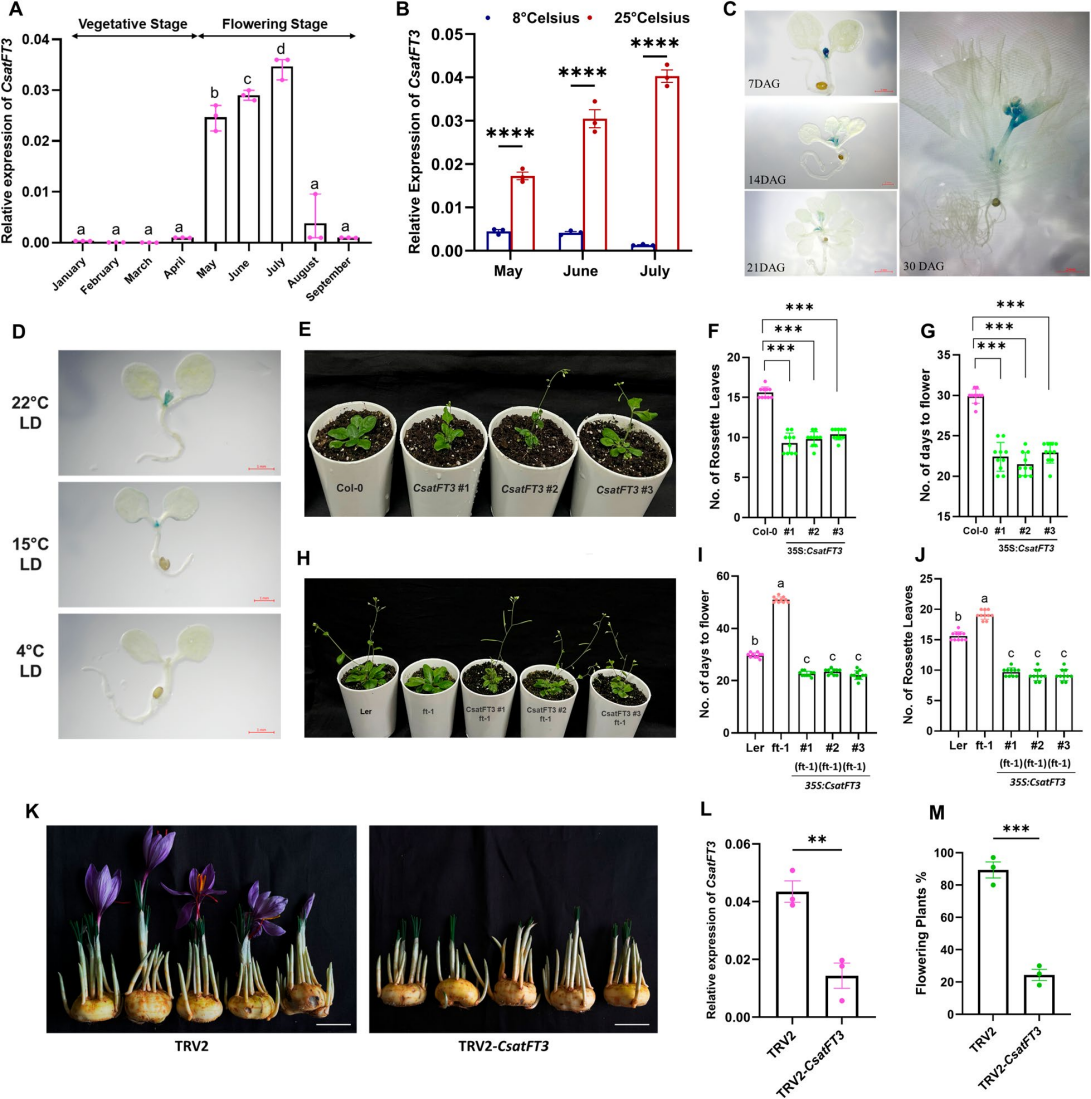
*CsatFT3* is involved in temperature‐mediated floral induction in saffron. (A) *CsatFT3* transcript levels in apical buds across developmental stages show specific expression during the reproductive phase. (B) Relative expression of *CsatFT3* in apical buds of saffron corms stored at ambient (25°C) and low (8°C) temperatures. Data are means ± SEM from 3 biological replications, where *n* = 10 for each replicate. (C) Histochemical GUS assay of the *CsatFT3* promoter (pro*CsatFT3*) in transgenic Arabidopsis in long‐day conditions (LD) during various developmental stages. 1.2 kb sequence upstream of the *CsatFT3* translation start sites (pro*CsatFT3*) was fused with the uidA (GUS) reporter gene. Different developmental stages include Arabidopsis plants after 7 Days After Germination (DAG), 14 DAG, 21 DAG and 30 DAG. *FT3* expression is confined to meristem and inflorescence. (D) Temperature‐sensitive GUS activity at 3 different temperatures. The experiment was repeated three times independently with similar results. (E–G) Ectopic expression of *CsatFT3* in Arabidopsis resulted in an early‐flowering phenotype. Statistical significance was analysed by Student's *t*‐test (*p < 0.05). (H–J) Functional complementation of the *ft‐1* mutant with *CsatFT3* restores flowering. Representative line expressing the number of rosette leaves and the number of days to flower. Total leaf number was calculated by combining total rosette leaves. Data represent a minimum of 10 plants scored for each line. Statistical significance was analysed by Student's *t*‐test (*p < 0.05). (K) Representative flowering vs. non‐flowering phenotypes in control and *CsatFT3*‐silenced saffron corms. The scale bar represents 2 cm. (L) qRT‐PCR validation of *CsatFT3* transcript knockdown in saffron corm apices following VIGS (*n* = 3 technical replicates, 10 independent corms). Error bars are shown as Standard Error of Mean (SEM) of three technical repeats. (M) Percentage of flowering in VIGS‐silenced vs. TRV2 control corms. The data presented include the mean percentage of flowering, with error bars representing the Standard Error of Mean (SEM) of three technical repeats (*n* = 3) and for each replicate, 10 corms were used. All data in this figure are shown as mean ± SEM. Statistical significance for data in panels A, I, and J was analysed using one‐way ANOVA followed by Tukey's Honest Significant Difference (HSD) test for multiple comparisons. Different letters indicate statistically significant differences between groups at *p* < 0.05, according to CLD. Data in panel B are analysed using two‐way ANOVA to assess the effects of temperature, month, and their interaction, followed by Sidak's multiple comparisons test. Asterisks indicate significant differences between 8°C and 25°C within each month (****p < 0.0001). F, G, L and M are analysed using the Student's *t*‐test (*p < 0.05; **p < 0.01).

Our previous work identified several PEBP family genes implicated in flowering regulation in saffron, with *CsatFT3* emerging as a potential key regulator of floral induction (Kalia et al. 2023). However, that study was limited to spatial and temporal expression profiling during the reproductive phase transition. To further elucidate the functional role of *CsatFT3*, we investigated its expression dynamics across vegetative and reproductive phases in saffron apical buds. We found that *CsatFT3* is specifically expressed during the reproductive phase, with transcript accumulation coinciding with the floral induction stage (Figure 1A, Figure S1). In contrast, its expression was barely detectable in apices of newly developing corms and during the vegetative phase in winter. Given that low temperatures are known to suppress floral induction in saffron (Jose‐Santhi et al. 2023), we next assessed *CsatFT3* expression in apical buds of corms stored under low (8°C) and ambient (25°C) temperature conditions following the vegetative phase. Consistent with a temperature‐responsive role, *CsatFT3* transcript levels were markedly reduced in corms stored at 8°C compared to those maintained at 25°C (Figure 1B).

To further investigate tissue‐ and temperature‐specific expression of the *CsatFT3* gene, we isolated a 1.2 kb fragment of the *CsatFT3* promoter (Figure S2) that contains several core, distal and proximal promoter elements and constructed a promoter‐GUS fusion construct. GUS‐driven transcription reporter lines of *CsatFT3* displayed meristem and flower‐specific expression in Arabidopsis, consistent with our spatiotemporal expression profiling in saffron (Figure 1C and Figure S3). Furthermore, these *ProCsatFT3::GUS* fusion lines demonstrated temperature‐sensitive regulation, with expression gradually decreasing as the temperature was lowered, in line with our previous findings that low temperatures suppress the expression of *CsatFT3* (Figure 1D). At low temperatures (4°C), GUS activity was nearly undetectable, indicating a significant suppression of expression under cold conditions. These findings reinforce the role of *CsatFT3* in temperature‐sensitive flowering regulation in saffron and suggest that its expression is activated under the relatively high‐temperature conditions that promote floral induction.

To functionally validate the role of *CsatFT3* in floral regulation, we ectopically expressed *CsatFT3* in Arabidopsis Col‐0 and complemented the late‐flowering *ft‐1* mutant (Ler‐0). Overexpression in Col‐0 resulted in a significant early‐flowering phenotype (Figure 1E–G), while *ft‐1* plants transformed with *CsatFT3* exhibited restored or even earlier flowering compared to wild‐type (Figure 1H–J). These results confirm that *CsatFT3* is functionally conserved and capable of promoting flowering in a heterologous system. Following confirmation of *CsatFT3's* involvement in floral induction and flowering, finally, to evaluate the role of *CsatFT3* in its native environment, we performed Virus‐Induced Gene Silencing (VIGS) of *CsatFT3* in saffron corms and monitored flowering. Silencing of *CsatFT3* led to a significant reduction in its transcript levels in apical buds (Figure 1K) and was accompanied by a marked decrease in flowering percentage compared to TRV2 mock‐infected controls (Figure 1L,M). No significant decrease of *CsatFT1* and *CsatFT2* was observed in TRV2‐silenced *CsatFT3* corms (Figure S4). Together, these findings establish *CsatFT3* as a temperature‐sensitive floral inducer in saffron, functioning as a key regulatory component during the transition from vegetative to reproductive growth.

### 3.2 Identification of Florigen Activation Complex Component FD, Involved in Flowering Induction in Saffron

*FT‐like* genes require interaction with the bZIP transcription factor‐*FD* to form the FT–FD complex that directly activates the floral meristem identity genes. Thus, to identify the FD‐like genes involved in floral induction of saffron, we have cloned full‐length cDNAs for three FD‐like genes (*CsatFD1*, *CsatFD2* and *CsatFD3*), which encode for 197, 215 and 245 aa of proteins, respectively. All three FDs are similar to FD proteins from other plants and contain the conserved basic region, leucine zipper region, and the functionally important conserved threonine (T)/SAP motif at the C terminus (Figure S5A). Phylogeny aligns CsatFD's with monocot FDs, with *Asparagus officinalis* as the nearest family member (Figure S5B).

**FIGURE 2:**
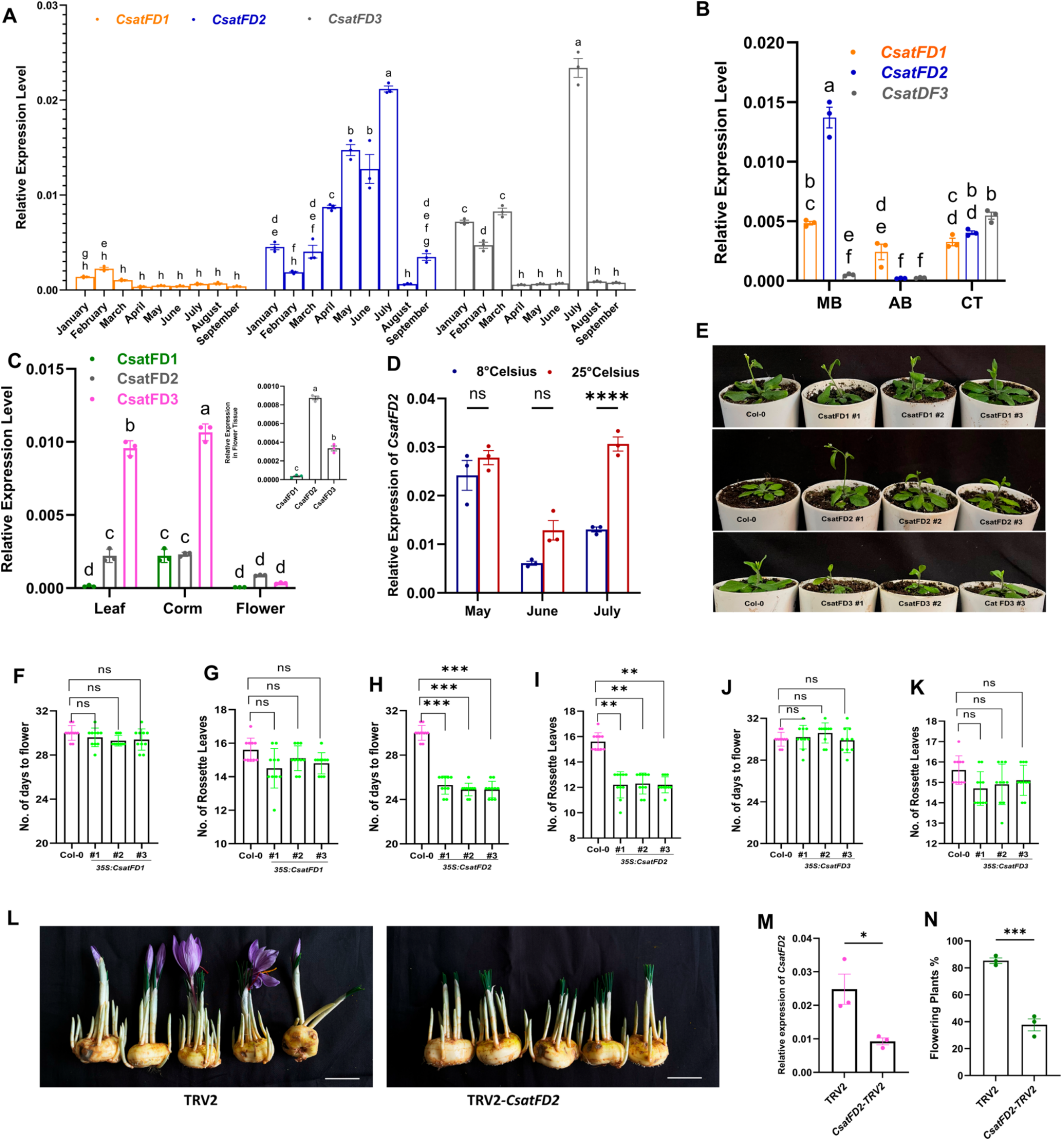
Functional characterisation of FD‐like genes in saffron. (A) Expression profiles of *CsatFD1*, *CsatFD2* and *CsatFD3* during vegetative and floral induction stages in the apical bud by RT‐qPCR. Data are means ± SE of 3 independent replicates. (B) Expression patterns of *CsatFD1*, *CsatFD2* and *CsatFD3* in the main bud (MB), axillary bud (AB) and corm tissue (CT) during the floral induction stage. Values are means ± SE of 3 independent replicates. (C) Tissue‐specific expression of FDs in different parts of the saffron plant. (D) Expression analyses of *CsatFD* genes in apical buds of corms stored at 25°C and 8°C during the months of May and June. (E) Phenotypic comparison of early flowering in *CsatFD2*‐expressing Arabidopsis compared to control. (F–K) Measurements of days to flowering and rosette leaf number in *CsatFD1*, *CsatFD2* and *CsatFD3* expressing Arabidopsis plants. Values are means ± SE (*n* = 10). Asterisks indicate a significant difference between the transgenic Arabidopsis lines and wild‐type plants (Col‐0; Student's *t*‐test, *p* < 0.05). (L) Phenotypes of *CsatFD2*‐TRV2 silenced lines and TRV2 lines. The scale bar represents 2 cm. (M) qRT‐PCR validation of *CsatFD2* knockdown in saffron corm apices after VIGS (*n* = 3). Error bars are shown as SD of three technical repeats. (N) Flowering percentage in TRV2 control and *CsatFD2* VIGS‐silenced saffron corms. The data presented include the mean percentage of flowering, with error bars representing the standard error of the mean (SEM) of three technical repeats (*n* = 3) and for each replicate, 10 corms were used. All data in this figure are shown as mean ± SEM. Statistical significance for data in panels A and B is analysed using two‐way ANOVA followed by Tukey's Honest Significant Difference (HSD) test for multiple comparisons. Different letters indicate statistically significant differences between groups at *p* < 0.05, according to CLD. Data in panel D are analysed using two‐way ANOVA to assess the effects of temperature, month and their interaction, followed by Sidak's multiple comparisons test. Asterisks indicate significant differences between 8°C and 25°C within each month (****p < 0.0001), F–K, M and N are analysed using the Student's *t*‐test (*p < 0.05; **p < 0.01).

We next investigated the expression profiles of the three saffron *FD* genes during the vegetative and flowering induction stages. Interestingly, the expression of *CsatFD2* coincided with the floral induction stage, whereas *CsatFD3* showed higher expression at later stages of sprouting, specifically in July, when floral differentiation and vegetative growth begin (Figure 2A). In contrast, *CsatFD1* was expressed at relatively stable levels throughout flower initiation and the vegetative phase, but its expression was generally lower compared to *CsatFD2* and *CsatFD3*. In saffron, the apical bud gives rise to the flower, while the axillary buds contribute only to vegetative growth, thereby distinguishing the reproductive meristem from the vegetative meristems (Kalia et al. 2022). Notably, *CsatFD2* was predominantly expressed in apical buds and flower tissues, indicating a primary role in flowering (Figure 2B,C). Additionally, *CsatFD2* showed comparatively higher expression in flower tissues compared to the other two *FD* genes (Figure 2C). We also examined the temperature‐sensitive expression of these genes in corms stored at low (8°C) and ambient high (25°C) temperatures. Our results revealed that *CsatFD2* expression was significantly downregulated at low temperatures (Figure 2D), whereas *CsatFD1* and *CsatFD3* did not show a similar trend (Figure S6A). Notably, *CsatFD1* expression remained unchanged at 8°C in June, the period when floral induction typically occurs. Interestingly, *CsatFD3* showed higher expression in corms stored at low temperatures compared to those stored at 25°C (Figure S6B).

Next, we examined the effect of ectopic expression of the saffron *FD* genes in Arabidopsis. Ectopic expression of *CsatFD2* can only induce early flowering in Arabidopsis, whereas there was no significant phenotypic change observed in *CsatFD1‐* and *CsatFD3*‐expressing Arabidopsis plants (Figure 2E). These findings were further supported by measurements of days to flowering and the number of rosette leaves in the Arabidopsis transformants (Figure 2F–K). These results further suggest that *CsatFD2* may be the primary gene involved in flowering regulation in saffron. To confirm the role of *CsatFD2* during floral induction, we silenced the gene using Virus‐Induced Gene Silencing (VIGS) in flower‐competent saffron corms. Compared to the TRV2 control, silencing of *CsatFD2* resulted in a reduction in flower formation and overall flowering percentage (Figure 2L). VIGS silencing of *CsatFD2* also led to a significant decrease in its transcript levels (Figure 2M) but did not affect the expression of *CsatFD1* and *CsatFD3* (Figure S7A,B). These results suggest that *CsatFD2* is a key component of the Floral Activator Complex (FAC) involved in floral induction in saffron.

### 3.3 TFL1‐3/ *CEN1* is a Newly Identified Negative Regulator of Flowering Induction in Saffron

**FIGURE 3:**
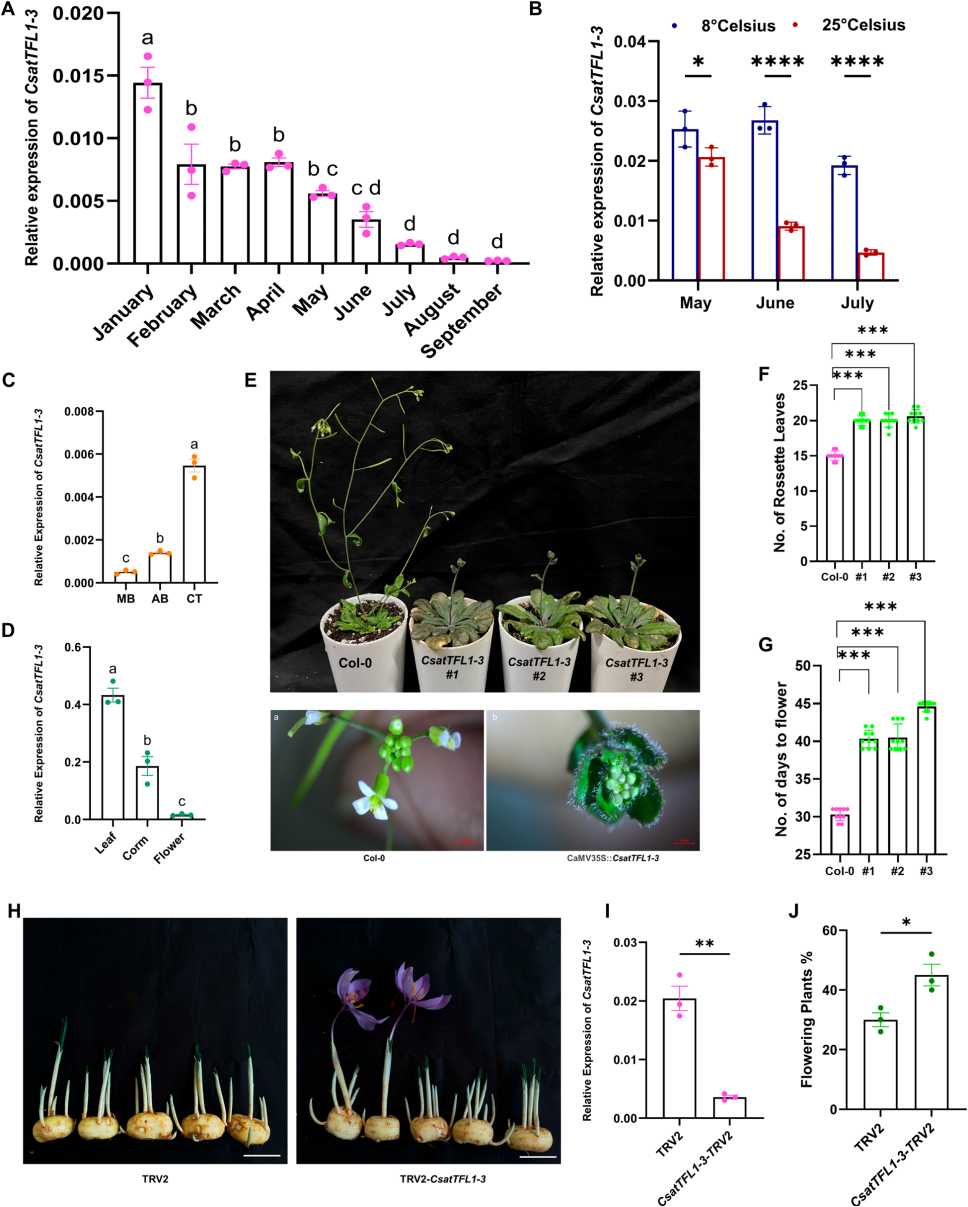
*CsatTFL1‐3* is a negative regulator of floral induction in saffron. (A) Relative expression of *CsatTFL1‐3* during the vegetative and reproductive phases in the apical bud by RT‐qPCR. Data are means ± SE of 3 independent replicates. (B) Expression analyses of the *CsatTFL1‐3* gene in apical buds of corms stored at 25°C and 8°C during floral induction time (C) Corm tissue‐specific expression of *TFL1‐3* during floral induction. (D) Tissue‐Specific Expression of *CsatTFL1‐3*. (E) Phenotypic analysis of Arabidopsis Col‐0 plants ectopically expressing *TFL1‐3*/*CEN1*: hyper vegetative phase phenotype was observed in *CsatTFL1‐3* transgenic lines of Arabidopsis. (F and G) Rosette leaf number and number of days to flower of 35S::*TFL1‐3* transgenic and control plants, respectively. Values are means ± SEM (*n* = 10). Asterisks indicate a significant difference between the transgenic Arabidopsis lines and wild‐type plants (Col‐0; Student's *t*‐test, *p* < 0.05). (H) Virus‐Induced Gene Silencing (VIGS) of *TFL1‐3*/*CEN1* in low‐flower‐competent saffron corms. The scale bar represents 2 cm. (I) The expression of *CsatTFL1‐3* in *CsatTFL1‐3*‐TRV2 silenced lines and TRV2 lines (*n* = 3). (J) Comparison of flowering percentages in VIGS‐silenced versus control saffron corms. The data presented include the mean percentage of flowering of three technical repeats (*n* = 3), and for each replicate, 10 corms were used. All data in this figure are shown as mean ± SEM. Statistical significance for data in panels A, C & D is analysed using one‐way ANOVA followed by Tukey's Honest Significant Difference (HSD) test for multiple comparisons. Different letters indicate statistically significant differences between groups at *p* < 0.05, according to CLD. Data in panel B are analysed using two‐way ANOVA to assess the effects of temperature, month and their interaction, followed by Sidak's multiple comparisons test. Asterisks indicate significant differences between 8°C and 25°C within each month (****p < 0.0001). F,G,I, & J are analysed using the Student's *t*‐test (*p < 0.05; **p < 0.01).

In our previous study (Kalia et al. 2023), we identified two homologues of *TFL‐1*/*CEN*, named Csat*TFL1‐1* and Csat*TFL1‐2*, and through spatiotemporal expression profiling, we proposed that *TFL1‐2* may play a role in determining flowering competency in saffron (Figure S8A,B). However, neither of these two *TFL‐1* genes exhibited a temperature‐sensitive response (Figure S9A,B). Furthermore, ectopic expression of *CsatTFL1‐1* and *CsatTFL1‐2* in Arabidopsis led to delayed bolting in only *CsatTFL1‐2* plants compared to wild‐type (WT) under long‐day (LD) conditions, whereas *CsatTFL1‐1* transformants bolted normally, similar to WT plants (Figure S10A–F). Additionally, interaction studies, including BiFC and Y2H assays, did not reveal any positive interactions with *CsatFD2*, our identified positive regulator of flowering (Figure S11A,B). These findings prompted us to investigate further, hypothesising that another homologue of *TFL1*/*CEN* might be involved in floral induction in saffron. Through corm development studies in our lab (Jose‐Santhi et al. 2023), we identified a third *TFL1* homologue, which exhibited higher expression during the vegetative phase. This gene shares similarities with *CsatTFL1‐1* and *CsatTFL1‐2*, but phylogenetic analysis clustered it within the *CEN* subgroup. Based on this, we named it *CsatTFL1‐3*/*CEN1* (Figure S12A,B). Like other *TFL1*/*CEN* family members, *TFL1‐3*/*CEN1* contains the conserved domains characteristic of this gene family. Quantitative PCR (qPCR) expression profiling revealed that *CsatTFL1‐3*/*CEN1* expression is elevated during the vegetative phase, with a significant decrease during the reproductive phase (Figure 3A). Tissue‐specific expression analysis confirmed that *CsatTFL1‐3*/*CEN1* is predominantly expressed in the axillary bud tissue, further supporting its potential role in negatively regulating flowering (Figure 3C,D).

Next, we examined the expression of *CsatTFL1‐3*/*CEN1* in corms stored at low (8°C) and ambient (25°C) temperatures. Interestingly, there was no reduction in *TFL1‐3*/*CEN1* expression in corms stored at low temperatures compared to those stored at ambient high temperatures, suggesting that it may be regulated by temperature and could play a role in temperature‐mediated floral induction in saffron (Figure 3B). We then performed ectopic expression of *CsatTFL1‐3*/*CEN1* in *A. thaliana* (Col‐0) under the control of the constitutive 35S promoter. Transgenic lines exhibited a significant delay in flowering compared to wild‐type plants, as evidenced by increased days to bolting and a higher number of rosette leaves at flowering (Figure 3F,G). These results confirm the ability of *TFL1‐3*/*CEN1* to act as a floral repressor in Arabidopsis, consistent with its proposed function in saffron. Notably, ectopic expression of *TFL1‐3*/*CEN1* also led to a hyper‐vegetative shoot phenotype, characterised by the development of an inflorescence composed of leaf primordia surrounded by small, serrated leaves—a phenotype not observed in *TFL1‐1* or *TFL1‐2* expressing Arabidopsis lines (Figure 3E). Finally, we performed Virus‐Induced Gene Silencing (VIGS) of *CsatTFL1‐3*/*CEN1* in low‐flower‐competent saffron corms. In comparison to TRV2 control plants, silencing of *CsatTFL1‐3*/*CEN1* led to the formation of floral structures in 7 to 9 g corms (classified as low‐flower‐competent), and these flowers appeared earlier than in TRV2 controls (Figure 3G–I). Importantly, VIGS of *CsatTFL1‐3*/*CEN1* did not affect the transcript levels of the other two *TFL1* homologues, *TFL1‐1* and *TFL1‐2* (Figure S13A,B), suggesting *TFL1‐3*/*CEN1* and not *TFL1‐1* or *TFL1‐2* could play a role in flowering in saffron. This observation suggests that *TFL1‐3*/*CEN1* acts as a negative regulator of floral induction, and its silencing promotes floral transition in otherwise low‐flowering competence corms.

### 3.4 The FT–FD–TFL Complex Involved in Floral Regulation

**FIGURE 4:**
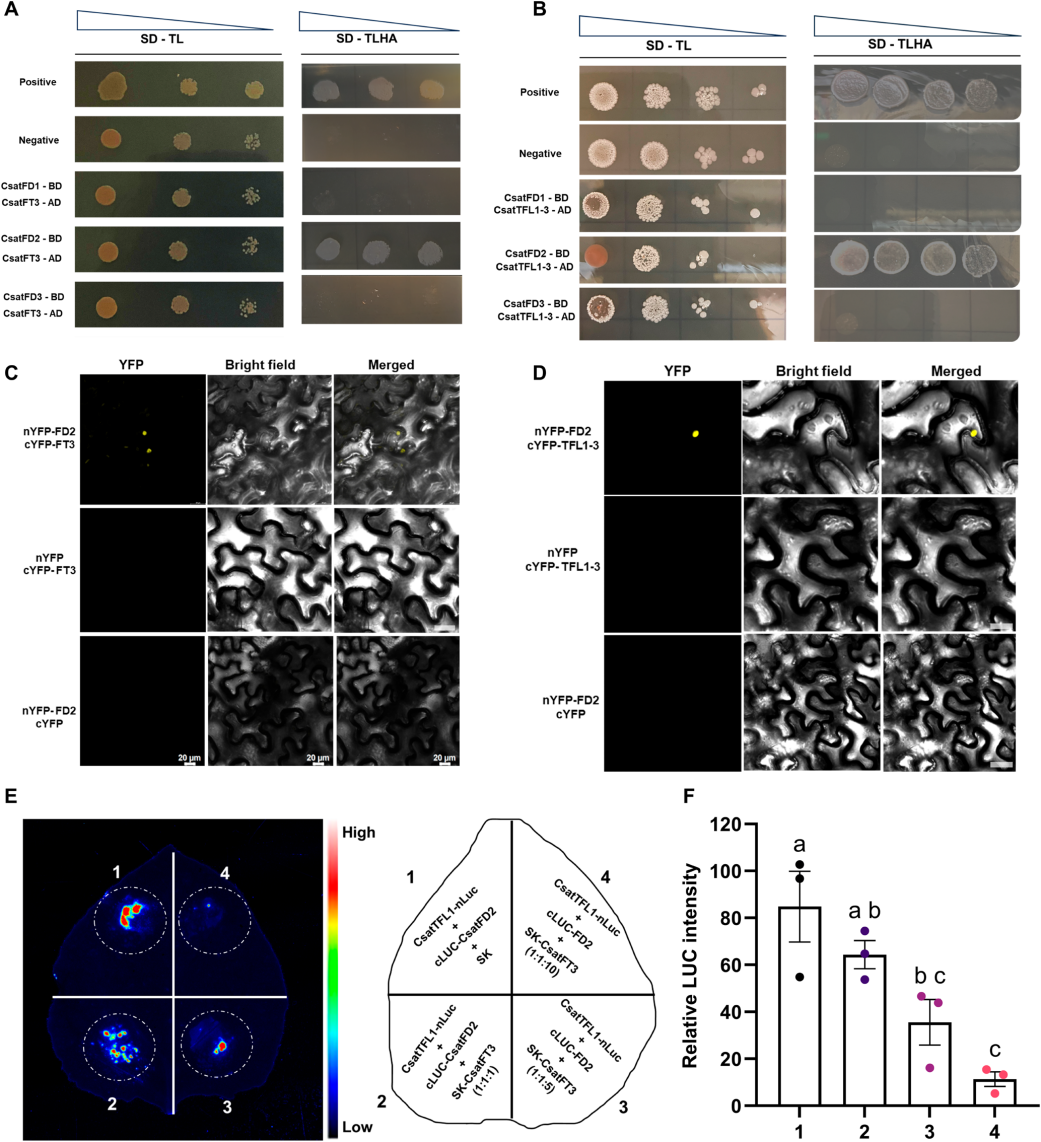
Interaction analysis of CsatFT3, CsatTFL1‐3 and CsatFD2. (A, B) Yeast two‐hybrid (Y2H) assays showing physical interactions between CsatFT3/CsatTFL1‐3 and CsatFD2. The coding sequences of *CsatFT3* and *CsatTFL1‐3* were fused to the activation domain in the pGADT7 vector, and *CsatFD2* was fused to the DNA‐binding domain in the pGBKT7 vector. Yeast transformants were grown on double dropout (DDO) medium lacking tryptophan and leucine (selection for transformation) and quadruple dropout (QDO) medium lacking tryptophan, leucine, histidine and adenine (selection for interaction). pGBKT7‐53 and pGADT7‐T were used as positive controls; pGBKT7‐lam and pGADT7‐T interactions served as the negative control. (C, D) Bimolecular fluorescence complementation (BiFC) assay showing nuclear localisation of the interactions between CsatFT3 or CsatTFL1‐3 with CsatFD2 in *N. benthamiana* leaf epidermal cells. CsatFD2 was fused to the C‐terminal half of YFP (cYFP), and CsatFT3 or CsatTFL1‐3 were fused to the N‐terminal half of YFP (nYFP). YFP signal indicates interaction. Bars = 20 μm. (E) Competitive luciferase complementation assay demonstrating the competition between CsatFT3 and CsatTFL1‐3 for binding to CsatFD2 in *N. benthamiana* leaves. *CsatTFL1* was fused to the N‐terminal half of luciferase (nLUC), and *CsatFD2* to the C‐terminal half (cLUC). Increasing amounts of *CsatFT3* (non‐tagged, driven by the 35S promoter, in the SK vector) were co‐infiltrated at different ratios. Images represent three independent replications. (F) Relative luminescence intensities indicate competition for *FD2* binding with *TFL1‐3* and *FT3*. Data represented is the mean of three replicates. Statistical significance is analysed using one‐way ANOVA followed by Tukey's Honest Significant Difference (HSD) test for multiple comparisons. Different letters indicate statistically significant differences between groups at *p* < 0.05, according to CLD.

Our earlier analyses identified *CsatFT3*, *CsatFD2* and *CsatTFL1‐3*/*CEN1* as potential core components of a Floral Activator Complex (FAC) in saffron. To investigate whether these proteins physically interact to form functional regulatory complexes, we employed two complementary approaches: yeast two‐hybrid (Y2H) assays and bimolecular fluorescence complementation (BiFC) in *N. benthamiana*. In the Y2H assays, the CDS of the three *CsatFD* genes were cloned into the pGADT7 prey vector and co‐transformed into yeast with either *CsatFT3* or *CsatTFL1‐3*/*CEN1* fused to the pGBKT7 bait vector. Interaction screening revealed that both *CsatFT3* and *TFL1‐3*/*CEN1* specifically interacted with *CsatFD2*, but not with *CsatFD1* or *CsatFD3* (Figure 4A,B). These results suggest that *CsatFD2* may serve as a central interacting hub for both activator (*FT3*) and repressor (*TFL1‐3*) proteins within the FAC.

We further validated these interactions by BiFC assays in *N. benthamiana* leaves. YFP fluorescence was observed in nuclei of cells co‐expressing the C‐terminal half of YFP fused to *CsatFD2* (cYFP–CsatFD2) with the N‐terminal half of YFP fused to either *CsatFT3* (nYFP–CsatFT3) or *CsatTFL1‐3* (nYFP–CsatTFL1‐3) (Figure 4C,D). No YFP fluorescence was detected in negative controls, including co‐expression of cYFP–CsatFD2 with nYFP alone or cYFP alone with nYFP–CsatFT3 or CsatTFL1‐3. Furthermore, consistent with the Y2H results, no BiFC signal was observed when *CsatFT3* or *TFL1‐3*/*CEN1* were co‐expressed with *CsatFD1* or *CsatFD3* (Figures S1–S18). Together, these findings strongly support a model in which *CsatFD2* specifically interacts with both *CsatFT3* and *TFL1‐3*/*CEN1*, suggesting that competitive complex formation may modulate floral transition in saffron.

Given the antagonistic roles of *CsatFT3* and *CsatTFL1‐3* in the regulation of flowering and their respective interactions with the transcription factor *CsatFD2*, it was hypothesised that CsatFT3 and CsatTFL1‐3 may compete for binding to CsatFD2. To investigate this, a luciferase complementation imaging (LCI) assay was conducted in *N. benthamiana* leaves to evaluate the interaction strength between CsatFD2‐nLUC and cLUC‐CsatTFL1‐3 in the presence or absence of SK‐CsatFT3. Strong luciferase (LUC) activity was observed when CsatFD2‐nLUC and cLUC‐CsatTFL1‐3 were coexpressed, indicating a robust interaction. However, co‐expression with SK‐CsatFT3 significantly reduced LUC activity, suggesting that CsatFT3 interferes with the interaction between CsatTFL1‐3 and CsatFD2. Moreover, this suppression was dose‐dependent, with increasing amounts of *CsatFT3* leading to a progressive decrease in LUC signal (Figure 4E). Biological replicates supporting these observations are presented in Figure S15A,B. These results strongly suggest that CsatFT3 competes with CsatTFL1‐3 for interaction with CsatFD2, which interferes with CsatTFL1‐3's ability to interact with CsatFD2.

### 3.5 *CsatSVP2* Acts Upstream of *FT3* in Temperature‐Mediated Floral Repression

To identify upstream genes potentially involved in temperature‐dependent floral induction in saffron, we screened transcriptome data representing the suppression of floral induction under low temperatures (Jose‐Santhi et al. 2023) and identified two partial SVP‐like MADS‐box genes that were upregulated in non‐flowering samples. *SVP*‐like genes are known to regulate flowering in a temperature‐dependent manner (Lee et al. 2007). Using long‐read and other in‐house transcriptome data, and based on sequence similarity, we obtained full‐length sequences of these two *SVP*‐like genes. We isolated and cloned them from saffron, naming them *CsatSVP1* and *CsatSVP2*. Sequence analysis indicated that while these genes are highly similar, they code for different amino acid sequences. Multiple sequence alignment revealed that both *CsatSVP1* and *CsatSVP2* contain the conserved MADS‐box domain, and phylogenetic analysis grouped them with *SVP*‐like genes from other geophytes, such as Narcissus, Zingiber and Lilium (Figure S16A,B).

**FIGURE 5:**
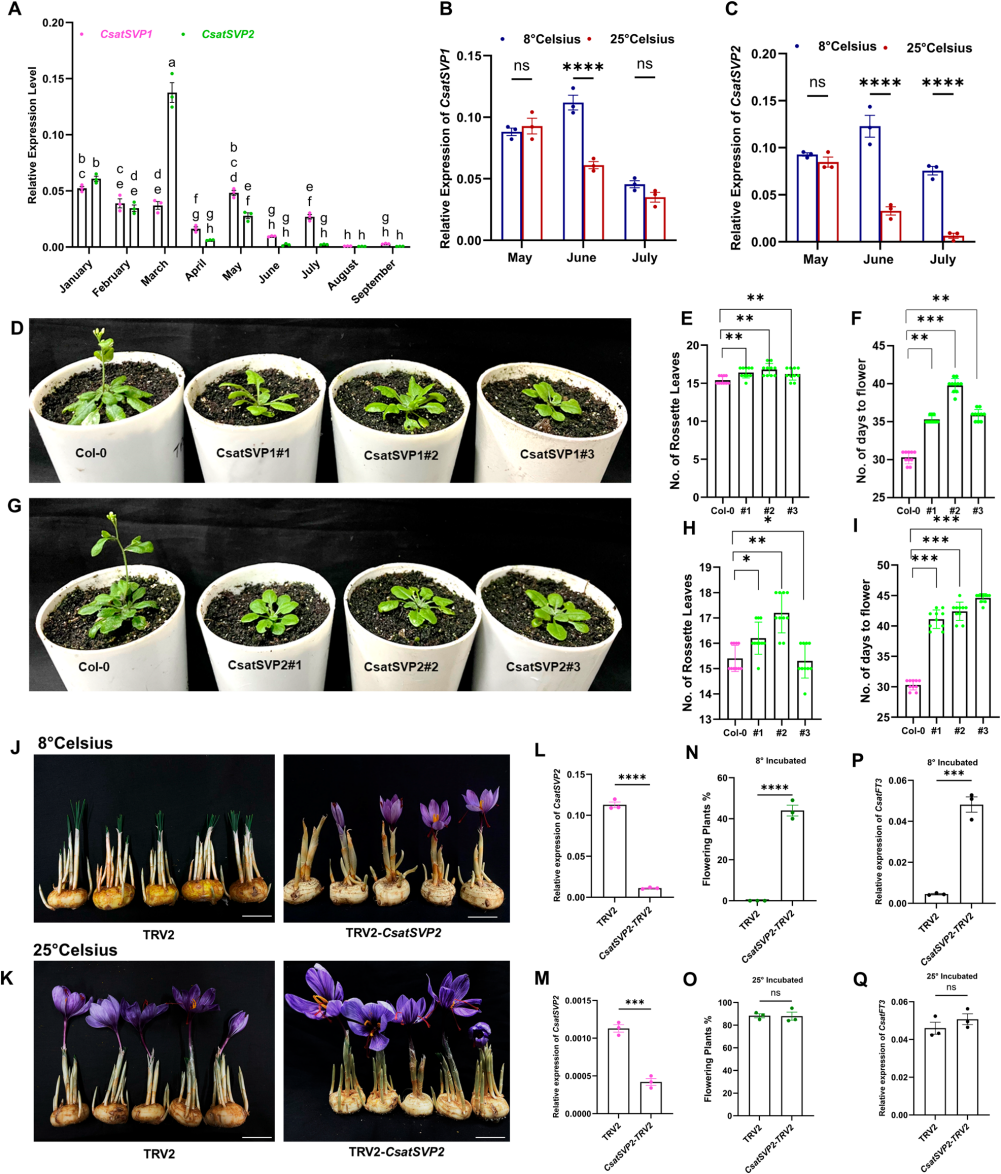
Functional characterisation of *CsatSVP1* and *CsatSVP2* in temperature‐dependent floral regulation. (A) Expression profiling of *CsatSVP1* and *CsatSVP2* in the apical bud during different vegetative and flowering stages. Data are means ± SE from 3 replications. (B and C) Expression of *SVP1* and *SVP2* genes in corms stored at inductive (25°C) and non‐inductive (8°C) temperatures, respectively. (D) Flowering phenotypes of Arabidopsis lines ectopically expressing *CsatSVP1*. (E, F) Rosette leaf number and number of days to flower of *CsatSVP1* transgenic and control plants, respectively. (G) Phenotypic changes of the floral organs in 35S::*CsatSVP2* transgenic Arabidopsis plants. (H, I) Rosette leaf number and number of days to flower of CsatSVP2 transgenic and control plants, respectively. The total leaf number was calculated by combining total rosette leaves. (J, K) Silencing of *CsatSVP*2 by VIGS rescues flowering under low‐temperature conditions in saffron. The scale bar represents 2 cm. (L, M) qRT‐PCR validation of *CsatSVP2* knockdown in saffron corm apices after VIGS stored at different temperatures (*n* = 3). Error bars are shown as SD of three technical repeats. (N, O) Flowering percentage in TRV2 control and *CsatSVP2* VIGS‐silenced saffron corms stored at low (8°C) and ambient (23°C) temperatures. The data presented include the mean percentage of flowering, with error bars representing the standard deviation (SD) of three technical repeats (*n* = 3), and for each replicate, 10 corms were used. (P, Q) Relative expression of *CsatFT3* in *SVP2*‐silenced versus control corms under different temperature regimes. All data in this figure are shown as mean ± SEM. Statistical significance for data in panel A are analysed using two‐way ANOVA followed by Tukey's Honest Significant Difference (HSD) test for multiple comparisons. Different letters indicate statistically significant differences between groups at *p* < 0.05, according to Compact Letter Display (CLD). Data in panels B and C are analysed using two‐way ANOVA to assess the effects of temperature, month and their interaction, followed by Sidak's multiple comparisons test. Asterisks indicate significant differences between 8°C and 25°C within each month (****p < 0.0001), E, F, H, I, L, M, N, O, P & Q are analysed using the student's *t*‐test (*p < 0.05; **p < 0.01).

To further characterise their role in flowering, we performed expression analysis of *CsatSVP1* and *CsatSVP2* during vegetative and floral induction stages. Both genes showed a significant reduction in expression during the floral induction stage (June); however, the reduction in *CsatSVP2* was more pronounced and correlated closely with floral induction and subsequent stages (Figure 5A). We then evaluated their expression in corms stored under floral inductive (25°C) and non‐inductive (8°C) conditions. *CsatSVP2* transcript levels remained high at both temperatures, suggesting it may contribute to the floral repression observed at low temperatures (Figure 5C). In contrast, *CsatSVP1* expression decreased under both conditions, showing no temperature‐dependent pattern (Figure 5B). Additionally, *CsatSVP2* expression was comparatively higher in floral tissues than *CsatSVP1*, further supporting its potential regulatory role (Figure S17).

Next, to functionally validate these genes, we ectopically expressed both *CsatSVP1* and *CsatSVP2* in Arabidopsis under a constitutive promoter. Ectopic expression of both genes resulted in delayed flowering, with a more pronounced delay in the CsatSVP2‐transformed lines compared to both the control and *CsatSVP1* lines (Figure 5D–I). Arabidopsis plants expressing *CsatSVP2* displayed various phenotypic changes in flower bud and flower morphology, producing smaller, more compact flowers and shorter siliques (Figure S18). To investigate the role of *SVP* in temperature‐mediated floral suppression, we silenced *CsatSVP2* in saffron corms using Virus‐Induced Gene Silencing (VIGS). Csat*SVP2* was selected due to its expression pattern and phenotype in Arabidopsis, which correlated with its expected function. Since low temperatures during storage or dormancy are known to suppress flowering, and *CsatSVP2* may be involved in this process, we stored the *CsatSVP2*‐silenced corms at low temperatures, alongside a mock control (TRV2). Silencing of *SVP2* resulted in flowering in corms at low temperatures, whereas the TRV2‐treated corms stored at low temperatures did not flower (Figure 5J). In parallel, corms stored at 25°C—an inductive condition—showed flowering in both control and *SVP2*‐silenced groups, confirming that *CsatSVP2* acts specifically in the floral suppression mechanism under cold conditions (Figure 5K). Gene expression analysis further revealed that *CsatSVP2* silencing prevented the typical low‐temperature‐mediated suppression of *CsatFT3* expression, thereby facilitating flowering (Figure 5P,Q). These findings suggest that *CsatSVP2* and *CsatFT3* act within the same regulatory pathway, mediating temperature‐responsive floral induction in saffron.

### 3.6 *SVP2* Directly Binds to the *CsatFT3* Promoter and Represses Its Expression

**FIGURE 6:**
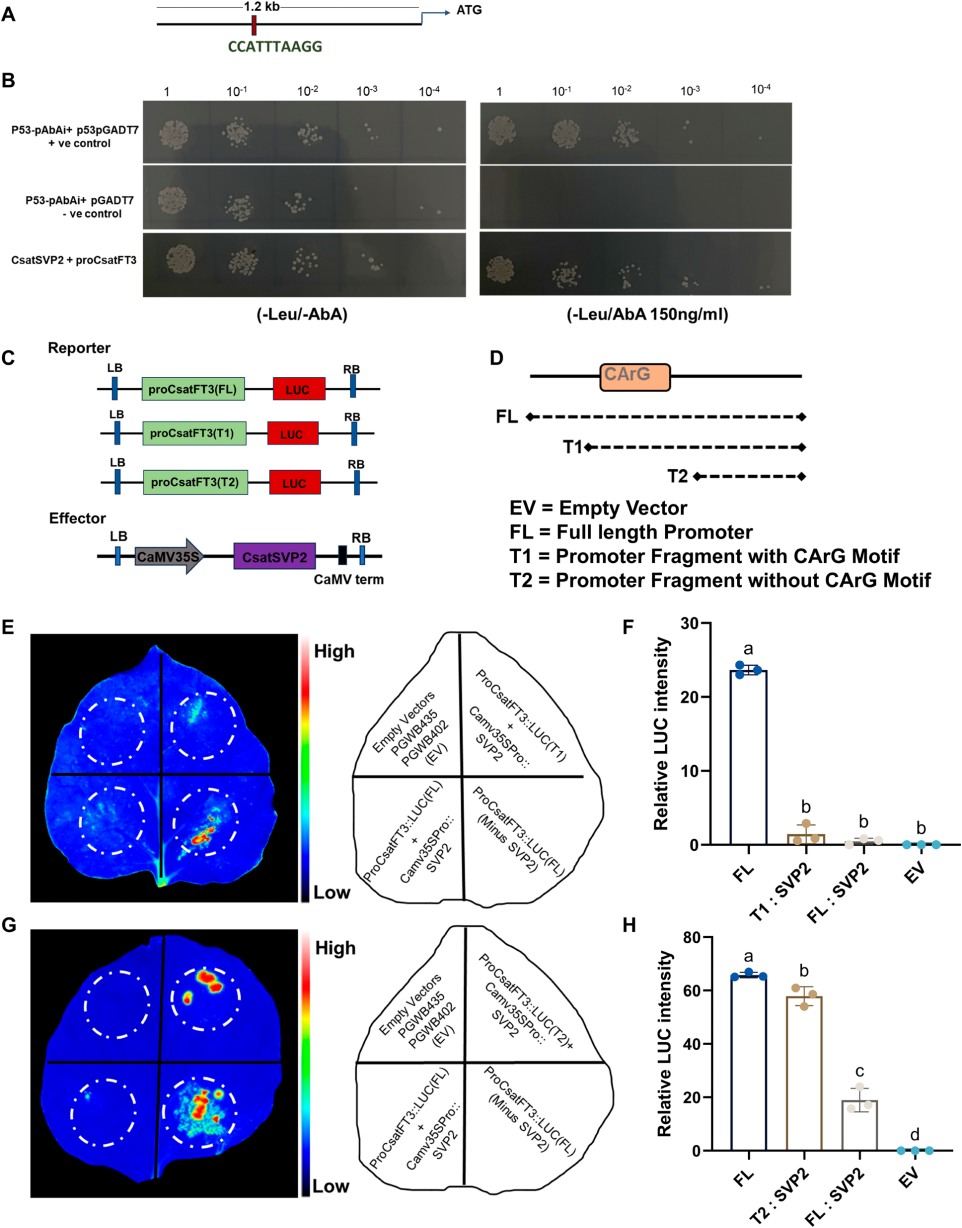
CsatSVP2 directly binds to the FT3 promoter via the CArG motif and represses its transcription. (A) Schematic diagram of the 1.2 kb *CsatFT3* promoter showing the location of a conserved CArG‐box motif (CCATTTAAGG) approximately 827 bp upstream of the ATG start codon. (B) Yeast one‐hybrid assay showing interaction between CsatSVP2 and the *FT3* promoter region containing the CArG motif. Growth on selective media confirms binding specificity. (C) Schematic diagram of the effectors and reporters used in dual‐luciferase reporter. (D) Schematic of the dual‐luciferase constructs used for transient expression in *N. benthamiana*: FL (full‐length promoter) T1 (with CArG motif) and T2 (without). (E) Relative luciferase activity in leaves co‐expressing *CsatSVP2* with the full promoter and T1 fragment construct of the *CsatFT3* gene. (F) Relative luciferase activity in leaves co‐expressing *CsatSVP2* and the *CsatFT3* full promoter and with T2 fragment constructs. Significant repression is observed only in the T1 construct, indicating that CsatSVP2 represses *CsatFT3* transcription through the CArG motif. (G, H) Quantification of luciferase activity. Bars represent mean ± SEM (*n* = 3). All data in the figure are shown as mean ± SEM. Statistical significance for data in panels F and H is analysed using one‐way ANOVA followed by Tukey's Honest Significant Difference (HSD) test for multiple comparisons. Different letters indicate statistically significant differences between groups at *p* < 0.05, according to Compact Letter Display (CLD).

Our previous results indicated that *CsatSVP2* and *CsatFT3* function in the same temperature‐sensitive regulatory pathway: low temperature maintains or upregulates *CsatSVP2* expression, which in turn suppresses *CsatFT3*, thereby repressing floral induction. Notably, *CsatFT3* transcript levels were restored in *CsatSVP2*‐silenced corms, suggesting that *CsatSVP2* functions upstream of CsatFT3. To further explore the mechanism of this regulation, we analysed the CsatFT3 promoter sequence to identify potential binding motifs for MADS‐box transcription factors. In silico analysis of the 1.2 kb *FT3* promoter, previously shown to drive temperature‐responsive expression, revealed a conserved CArG‐box motif (CCATTTAAGG) located approximately 827 bp upstream of the ATG start codon that is established for binding of MADS box genes, including *SVP* orthologs (Figure 6A). To experimentally test further whether *CsatSVP2* directly binds to the *CsatFT3* promoter, we performed a yeast one‐hybrid (Y1H) assay. The Y1H assay confirmed that *CsatSVP2* specifically binds to the *CsatFT3* promoter, supporting the hypothesis of direct regulation (Figure 6B). To further validate the functional specificity of this interaction, we employed a luciferase reporter assay using *N. benthamiana* transient expression (Figure 6C) and conducted a luciferase reporter assay using three different *FT3* promoter fragments: (1) the full‐length promoter (~1.2 kb), (2) a truncated version containing the conserved CArG motif (T1) and (3) a truncated version lacking the motif (T2) (Figure 6D). Co‐expression of *CsatSVP2* with these constructs in *N. benthamiana* revealed that luciferase activity was significantly reduced in both the full‐length and T1 constructs (Figure 6D,F). In contrast, no repression was observed with the T2 construct, confirming that SVP2 specifically suppresses *FT3* expression through direct binding to the CArG motif (Figure 6F). Together, these results establish a mechanistic model in which *CsatSVP2* acts as a temperature‐responsive floral repressor that directly binds to the *FT3* promoter via a conserved CArG motif, thereby repressing *FT3* expression and floral induction under low‐temperature conditions.

## 4 Discussion

Saffron is a high‐value crop cultivated in limited regions due to its strict temperature requirements, which distinctly regulate its vegetative and reproductive growth. High temperatures induce flowering, while subsequent cooling triggers flower formation. In contrast, low temperatures suppress floral induction and support vegetative growth, enabling overwintering and daughter corm development (Molina, Valero, Navarro, Guardiola, and Garcia‐Luis 2005). This temperature sensitivity restricts saffron cultivation to specific climates. Although environmental control of flowering has enabled cultivation in soilless and controlled settings, the molecular mechanisms underlying temperature‐regulated flowering remain poorly understood. As a sterile crop, saffron cannot be improved through conventional breeding, making biotechnological interventions essential. However, limited genomic and genetic resources, challenges in genetic transformation and a scarcity of molecular studies have hindered progress in functional genomics, creating a critical knowledge gap that must be addressed.

**FIGURE 7:**
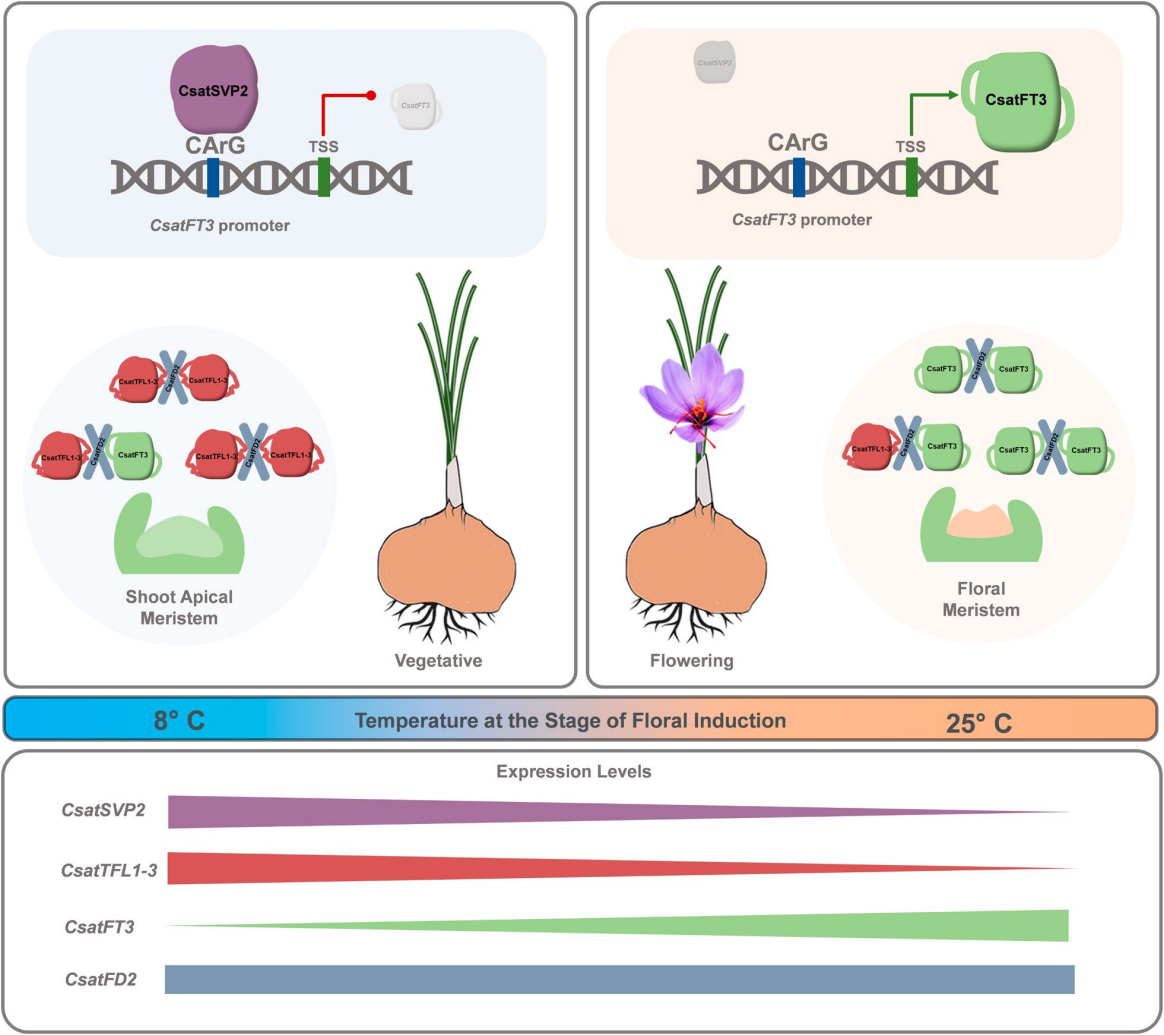
Proposed model of temperature‐mediated flowering regulation in saffron. This schematic model illustrates the temperature‐regulated floral induction pathway in saffron, emphasising the key roles of *CsatFT3*, *CsatFD2*, *CsatTFL1‐3*/*CEN1* and *CsatSVP2* in transitioning from vegetative to reproductive growth. At low temperatures (e.g., below 10°C), *CsatFT3* expression remains low, and CsatTFL1‐3/CEN1 binds to CsatFD2, preventing the formation of the Floral Activator Complex (FAC) and maintaining the shoot apical meristem in a vegetative state and inhibiting flowering. Additionally, *CsatSVP2* is upregulated at low temperatures and binds directly to a conserved CArG motif in the *CsatFT3* promoter, repressing its transcription and reinforcing the suppression of flowering. As temperatures increase beyond a critical threshold (e.g., above 25 C), *CsatFT3* expression is upregulated, enabling its interaction with CsatFD2 to form the FAC, which in turn activates floral meristem identity genes and promotes floral initiation. Concurrently, *TFL1‐3*/*CEN1* expression is downregulated, releasing the repression on *CsatFD2* and facilitating the transition to reproductive development.

In this study, we have identified a temperature‐sensitive transcriptional network that regulates flowering in saffron, uncovering both conserved and saffron‐specific components involved in floral induction (Figure 7). Our data establish a regulatory module involving *CsatFT3*, *CsatFD2*, *CsatTFL1‐3*/*CEN1* and *CsatSVP2* as key components in mediating floral transition in response to temperature cues. Although the core elements of the Florigen Activation Complex (FAC)–FT, *FD* and *TFL1* are conserved across angiosperms, their expression patterns, protein interactions and functional dynamics in saffron suggest neofunctionalisation in line with its geophytic lifecycle. For instance, notably, *CsatFT3* and *CsatFD2* are upregulated at higher temperatures and co‐expressed in the floral meristem, promoting flowering. In contrast, other FT and FD homologues show no such expression correlation (Figure 2; Kalia et al. 2022), indicating their limited or no involvement in floral induction. Similarly, CsatTFL1‐3 functions as a floral repressor by competitively binding to CsatFD2, whereas the other two TFL1 homologues neither interact with CsatFD2 nor delay flowering in Arabidopsis, suggesting a lack of functional redundancy and functional specificity. Functional validation in both Arabidopsis and saffron confirms that floral induction is governed by the dynamic interplay between CsatFT3, CsatFD2 and CsatTFL1‐3. Given the roles of gene duplication, neofunctionalisation and lineage‐specific gene expansion in driving the specialisation of reproductive and vegetative pathways in geophytes (Khosa et al. 2021), our identification of distinct regulatory factors and their molecular interactors from multigene families in saffron represents a significant advance.

A distinguishing feature of floral induction observed in saffron is the spatial restriction of flowering‐related gene expression predominantly to the meristem. All the genes identified in this study were specifically expressed in the apical (flowering) meristem. During floral induction, saffron corms are either underground or stored in the dark and remain leafless, indicating that the molecular signals initiating flowering are localised within the apical buds. Such meristem‐localised control may reflect an adaptation to the geophytic habit of saffron, which undergoes floral induction in the absence of foliage, similar to what has been observed in other geophytes such as tulips (Leeggangers et al. 2017). This localisation supports the hypothesis that temperature cues are sensed and interpreted locally within the corm, rather than systemically via leaves, as observed in many other species, including some geophytes (Lee, Baldwin, et al. 2013; Navarro et al. 2011; Wigge 2011; Wu et al. 2022; Yan et al. 2021).

Temperature plays a pivotal role in regulating flowering time across plant species, often through conserved thermosensory pathways. In Arabidopsis, warmer temperatures promote flowering via *FT* activation (Balasubramanian et al. 2006), while in crops like strawberry and onion, cold exposure induces flowering by triggering *FT*‐like genes (Heide 1977; Lee, Baldwin, et al. 2013). Interestingly, the effect of temperature on flowering is not always straightforward. Temperatures above 25°C seem to have both inductive and repressive effects on flowering, depending on the species and environmental conditions (Nakano et al. 2013; Noy‐Porat et al. 2013; Oda et al. 2012). This temperature sensitivity is closely linked to the regulation of *FT*, a key gene that integrates various environmental signals and controls the timing of flowering. In *Narcissus tazetta* (daffodils), floral induction is triggered by high ambient temperatures (25°C) through the activation of *NtFT*, while in tulips, *TgTFL1* downregulation and *TgFT2* upregulation contribute to temperature‐induced floral induction (Leeggangers et al. 2017; Noy‐Porat et al. 2013). In contrast, in species like onion and lily, which flower in spring, vernalisation (low‐temperature treatment) is required to activate the expression of *AcFT2* and *LiFT*, respectively (Lee, Baldwin, et al. 2013). In our study, we also found that *CsatFT3*, a key flowering‐time gene in saffron, is temperature‐regulated, with its expression being induced by high ambient temperatures and suppressed by low temperatures (Figure 1B). Similarly, *CsatFD2*, a partner of *CsatFT3* in the flowering regulatory network, follows a similar expression pattern, suggesting their involvement in the temperature‐mediated floral induction pathway in saffron. Interestingly, we observed that *CsatTFL1‐3*, a gene that maintains the vegetative state and prevents early flowering, exhibits a reciprocal expression pattern. In Arabidopsis, low and ambient temperature regulation of flowering involves the gene *TFL1* (Strasser et al. 2009). Higher expression of *CsatTFL1‐3* is observed during the vegetative phase, which coincides with the low winter temperatures during saffron's growth cycle (Figure 3A). This expression pattern is crucial for maintaining the vegetative phase and delaying flowering, as confirmed by the hyper‐vegetative shoot phenotype in *TFL1*‐3 overexpressing Arabidopsis plants (Figure 3) similar to what was observed when Arabidopsis *TFL1* is overexpressed (Lee et al. 2019). In contrast, as temperatures rise in the summer, the expression of *CsatFT3* increases (Figure 1A), likely triggering the activation of floral pathway genes and the transition to reproductive development. Overall, a balance between *CsatFT3*, *CsatTFL1‐3* and their interaction with *CsatFD2* governs floral induction in a temperature‐dependent manner. This delicate interplay between temperature and gene expression is vital for ensuring the proper timing of flowering in saffron, aligning the plant's reproductive phase with favourable environmental conditions.

We also identify *CsatSVP2*, a MADS‐box transcription factor, as a critical floral repressor acting upstream of the FT–FD–TFL core module. MADS‐box genes are key regulators of flowering time, with *SVP*‐like genes playing central roles in ambient temperature‐mediated floral control (Jin and Ahn 2021). CsatSVP2 directly binds a conserved CArG‐box in the *CsatFT3* promoter, repressing its expression under low temperatures. Silencing of *SVP2* alleviated low‐temperature‐induced flowering suppression and restored *CsatFT3* expression, thereby establishing a direct mechanistic link between ambient temperature perception and floral induction (Figure 5). In *A. thaliana* , a comparable regulatory pathway has been described, where SVP negatively regulates *FT* expression by directly binding to a CArG motif in its promoter (Lee et al. 2007). Additionally, *SVP* levels are known to increase under low temperatures and are degraded at higher temperatures, facilitating early flowering as *SVP* abundance declines (Blázquez et al. 2002; Lee, Ryu, et al. 2013; Sureshkumar et al. 2016). Interestingly, individual members of the SVP gene family have been shown to play distinct roles in processes such as bud dormancy, flowering initiation and floral development, reflecting adaptations of developmental timing to local environmental conditions and seasonal temperature fluctuations. In tulips, gene expression analyses have similarly identified *TgMADS15*/*16* and *TgMADS9* as low‐temperature‐responsive *SVP*‐like genes involved in floral induction (Lu et al. 2023), reinforcing the conserved role of this gene family in temperature‐mediated flowering regulation across geophytic species. *SVP* homologues in Lilium and *Narcissus tazetta* have also been reported as flowering repressors, though their temperature responsiveness remains unexplored (Li et al. 2015; Wu et al. 2024). Notably, ectopic expression of *CsatSVP2* in Arabidopsis caused floral morphology defects, reminiscent of phenotypes observed with Lilium SVP expression (Wu et al. 2024), suggesting that these homologues may also contribute to flower development in addition to regulating flowering time.

## 5 Conclusions

Collectively, our findings establish a detailed molecular framework for temperature‐regulated flowering in saffron, centred on the dynamic balance between floral activators (*CsatFT3*, *CsatFD2*) and repressors (*CsatTFL1‐3*, *CsatSVP2*). The spatiotemporal expression patterns and protein interactions among these components provide key insights into how flowering is precisely modulated in response to environmental temperature fluctuations. By uncovering saffron‐specific adaptations within otherwise conserved gene networks, this study advances our understanding of floral regulation in geophytes and sterile crops. Importantly, these results lay a foundation for the development of genetic and biotechnological strategies to improve saffron yield, synchronise flowering and enhance resilience to climate variability. Our work highlights a saffron‐specific model of thermoresponsive flowering that builds upon conserved floral regulatory pathways yet exhibits functional divergence shaped by the geophytic lifecycle. Beyond saffron, these findings offer a valuable framework for translational research in other temperature‐sensitive geophytes such as Tulip, Narcissus, Hyacinthus and Iris, where molecular insights remain scarce (Khodorova and Boitel‐Conti 2013). By identifying novel regulatory targets, this study opens new avenues for improving flowering and productivity in saffron and related high‐value geophytic species.

## Author Contributions

R.K.S. conceptualised the research idea and designed the work plan. D.K., J.J.S. and F.R.S. conducted the experiments and collected the data. D.K., J.J.S. and R.K.S. analysed the data and contributed to writing the manuscript. All authors read and approved the final version of the manuscript.

## Conflicts of Interest

The authors declare no conflicts of interest.

## Supporting information


**Data S1:** pbi70361‐sup‐0001‐Supinfo.docx.

## Data Availability

The data that support the findings of this study are available on request from the corresponding author.
